# Avenir® vs. Axium^TM^ Coils for the Treatment of Intracranial Aneurysms: Results of a Multicenter Randomized Controlled Trial With Short-Term Follow-Up

**DOI:** 10.3389/fneur.2021.817989

**Published:** 2022-01-26

**Authors:** Wei Li, Ming Ye, Alexandru Cimpoca, Hans Henkes, Honglei Wang, Xiang Xu, Yuxiang Gu, Huaizhang Shi, Hongming Ji, Feng Wang, Yuanli Zhao, Geng Guo, Hongqi Zhang, Youxiang Li

**Affiliations:** ^1^Department of Interventional Neuroradiology, Beijing Neurosurgical Institute, Beijing Neurosurgical Institute, Capital Medical University, Beijing, China; ^2^Neurosurgery Department, The Second Affiliated Hospital of Xingtai Medical College, Xingtai, China; ^3^Xuanwu Hospital of Capital Medical University, Beijing, China; ^4^Neuroradiological Clinic, Klinikum Stuttgart, Stuttgart, Germany; ^5^Medical Faculty, University Duisburg-Essen, Essen, Germany; ^6^The First Hospital of Jilin University, Jilin, China; ^7^Tangshan Worker's Hospital, Tangshan, China; ^8^Huashan Hospital of Fudan University, Beijing, China; ^9^The First Affiliated Hospital of Harbin Medical University, Harbin, China; ^10^Shanxi Provincial People's Hospital, Taiyuan, China; ^11^The First Affiliated Hospital of Dalian Medical University, Dalian, China; ^12^Peking University International Hospital, Beijing, China; ^13^The First Hospital of Shanxi Medical University, Taiyuan, China; ^14^Beijing Tiantan Hospital of Capital Medical University, Beijing, China

**Keywords:** intracranial aneurysm, endovascular procedures, coil occlusion, safety, comparative effectiveness research

## Abstract

**Purpose:**

Endovascular coil occlusion is a successful and rapidly evolving strategy used to treat patients who present with intracranial aneurysms. This study aimed to compare the safety and efficacy of the Avenir® and Axium^TM^ passive mechanically detachable coil systems.

**Methods:**

A prospective, multicenter, randomized controlled study was carried out at ten medical centers from March 2018 to December 2019. A series of consecutive patients diagnosed with intracranial aneurysms were randomly assigned to undergo endovascular treatment with either the Avenir® or the Axium^TM^ mechanically detachable coil systems. The short-term outcomes from the two groups were compared with a focus on treatment efficacy and safety.

**Results:**

A total of 162 and 161 patients were enrolled in the Avenir and Axium groups, respectively. The rate of successful coil detachment was 100% for the Avenir group and 99.38% for the Axium group. At the six-month follow-up visit, the overall aneurysm occlusion rate was 94.66% for the Avenir group and 96.95% for the Axium group (*p* > 0.05). We observed no statistically significant differences in clinical condition (as per the modified Rankin Scale) or the degree of aneurysm occlusion (as determined by digital subtraction angiography [DSA] and Raymond-Roy Occlusion Classification). Surgical complications were reported in 27 subjects in the Avenir group and 22 in the Axium group (*p* > 0.05). DSA performed at 6 months revealed complete aneurysm occlusion in 84 and 86% of patients in the Avenir and Axium groups, respectively.

**Conclusion:**

We observed no significant short-term differences with respect to efficacy or safety when using either Avenir® or Axium^TM^ coils for the treatment of intracranial aneurysms.

## Introduction

The incidence of intracranial aneurysms was reported to be at least 1–2% in an unselected adult population (1). Unruptured intracranial aneurysms can be found incidentally on imaging studies, or they may be heralded by symptoms that include headaches, hemiparesis, visual field defects, and seizures. As the aneurysms enlarge, they may compress adjacent neuronal structures. While only a small proportion of intracranial aneurysms will ultimately rupture, this complication can result in subarachnoid hemorrhage and a 30% risk of death or permanent disability (2, 3). There are currently three strategies that can be used to treat intracranial aneurysms that have ruptured or are at risk of doing so, including conservative management, endovascular treatment, or craniotomy with microsurgical clipping (4, 5). Endovascular techniques are gradually becoming the preferred first-line option as they are significantly less invasive than open surgery. When compared to microsurgical clipping, the use of endovascular techniques to treat incidental, symptomatic, or ruptured intracranial aneurysms has resulted in equivalent or improved clinical outcomes (6). Other studies confirmed that the use of endovascular strategies resulted in improved long-term outcomes for patients with ruptured lesions (7), although the clinical outcomes for coil occlusion and microsurgical clipping of unruptured aneurysms were virtually identical (8–10).

The two main complications of endovascular coil occlusion for intracranial aneurysms are peri-procedural perforation of the aneurysm and thromboembolic events. The standard endovascular procedure involves the insertion of a microcatheter and microguidewire to target the aneurysm followed by the insertion of a suitable coil which then undergoes controlled detachment. This procedure has evolved rapidly over the past 30 years (11). The long period that was initially required for detachment of the coil spurred several medical device manufacturers to develop novel methods to facilitate its rapid detachment under controlled conditions. The Target® electrolytically detachable coil system was first released in 1995, followed in 2002 by the Hydrolink hydraulic detachable coil device (MicroVention, Inc.) In 2005, MICRUS (now Codman Neurovascular, Inc.) introduced a thermoelectric detachable coil system. Medtronic PLC released a mechanically detachable coil system (Axium^TM^) in 2007 (12).

To date, there are only a few prospective studies that focus on the safety and efficacy of these endovascular coils. Among the most significant concerns, aneurysms may recur due to coil compaction. Likewise, a coil may migrate into an intra-aneurysmal thrombus and trigger modifications to its surface. Likewise, there is no evidence to suggest that the use of modified coils can significantly improve the long-term outcomes of endovascular procedures compared to the use of conventional platinum coils (13–15). Although bare platinum coils are currently in wide use, there remains considerable room for improvement. Coil occlusion of an aneurysm is a complex procedure, and the coils used must have reliable and consistent physical properties. Among these properties, coils must be easy to insert and be clearly visible, with consistent filling and a straightforward detachment mechanism to facilitate complete removal of the pushrod after the procedure (16, 17).

Coil systems that are currently available use different modes of energy transmission to achieve detachment, including electrical, hydraulic, and mechanical impact (12). Mechanically detachable coil systems are passive in nature, undergo rapid detachment, and require only a few supporting devices. The Avenir® coil system (Wallaby Medical, Shanghai, China) is a new, noose-based, passive mechanically-detachable coil system that needs no detachment tools.

This study aimed to evaluate the safety and efficacy of the Avenir® mechanically detachable coil system for the treatment of intracranial aneurysms in clinical practice.

## Materials and Methods

### Research Design and Subjects

This study was a prospective, multicenter, randomized controlled validation trial with a non-inferiority trial design. The study protocol was approved by the ethics committees of Beijing Tiantan Hospital of Capital Medical University (Institutional Review Board of Beijing Tiantan Hospital, Capital Medical University/ID approval number QX2017-006-03), Xuanwu Hospital of Capital Medical University (Institutional Review Board of Xuanwu Hospital Capital Medical University/ID approval number 2018-003), Huashan Hospital of Fudan University (Huashan Hospital Institutional Review Board/ID approval number 2018-474), the First Hospital of Jilin University (Institutional Review Board of First Hospital of Jilin University/ID approval number 19Q012-001), the First Affiliated Hospital of Dalian Medical University [Institutional Review Board of First Affiliated Hospital of Dalian Medical University/ID approval number PJ-JG-QX-2018-06(X)], the First Affiliated Hospital of Harbin Medical University (The Ethics Committee of First Affiliated Hospital of Harbin Medical University/ID approval number 201803), Shanxi Provincial People's Hospital (Institutional Review Board of Shanxi Provincial People's Hospital/ID approval number 2018-001), Peking University International Hospital (Institutional Review Board of Peking University International Hospital/ID approval number E2018-003), the First Hospital of Shanxi Medical University (Ethics Committee of First Hospital of Shanxi Medical University/ID approval number 2018-Q11), and Tangshan Worker's Hospital (Institutional Review Board of Tangshan Worker's Hospital/ID approval number 2018-06).

This clinical trial was conducted under Good Clinical Practice regulations. All patients were informed of the nature, purpose, and potential risks associated with the trial and each signed informed consent forms before participation. The trial passed the Shanghai Food and Drug Administration (FDA) inspection with no significant findings and also passed the clinical trial quality check for each participating hospital. The clinical trial registration number is ChiCTR2100046506.

Patients with intracranial aneurysms who presented at one of the ten participating hospitals between March 2018 and December 2019 were enrolled prospectively. Study institutions competed with one another to enroll patients from each of the different hospitals. Patient inclusion criteria included (1) males and females 18–80 years of age, (2) diagnosis of at least one intracranial aneurysm based on computed tomography angiography (CTA), magnetic resonance angiography (MRA), or digital subtraction angiography (DSA) imaging performed during the previous 6 months, (3) clinical condition rated as Hunt-Hess grade 0, I, II, or III (18), and (4) a clear understanding of the trial information and capacity to sign the informed consent form. Patients were excluded if (1) their condition precluded endovascular treatment of the aneurysm (e.g., due to a space-occupying effect of an intracranial hematoma, giant aneurysm), (2) they were diagnosed with liver or kidney dysfunction (defined as alanine aminotransferase [ALT] or aspartate transaminase [AST] levels that were more than two times greater than the upper limit of normal), (3) they had participated in other clinical trials in the past 3 months, (4) they were diagnosed with concomitant diseases that would preclude effective treatment or evaluation (e.g., cancer, infection, severe metabolic diseases, and/or mental disorders), (4) they were pregnant, or (5) they were allergic or had contraindications to aspirin, heparin, or local or general anesthesia.

### Random Grouping and Blinding Method

Patients were assigned at random to one of the two groups. To avoid bias, each patient was assigned to the Avenir or Axium group based on information they received after scratching off the silver coating on a random card that was provided in the order in which they were enrolled in the study. Complementary treatment was based on the group assignment determined by the random card distribution.

The patients assigned to the Avenir and Axium groups were evaluated using the same criteria. All imaging findings were assessed in a blinded manner by neurosurgeons or radiologists with an intermediate professional title or higher who were not directly involved in the clinical study or the data collection. Assessments were made several times for each patient. Means and medians were used to generate the final results.

### Intervention

Routine preoperative and postoperative assessments included coagulation tests (prothrombin time [PT] and activated partial thromboplastin time [APTT]) and platelet counts were performed for all patients. Results from a thromboelastography (TEG) procedure were assessed in patients with a bleeding tendency.

Quality assurance measures included: (1) the interventional procedure was performed by a high-level associate chief or neuro-interventionalist physician with more than 5 years of experience with independent aneurysm coil treatments in large tertiary care hospitals, and (2) all individuals performing the surgery had been trained with standardized procedures and methods before the initiation of the study.

All procedures were performed with the patient under general anesthesia. The procedure involved a femoral artery puncture followed by placement of a sheath using the Seldinger technique. All patients were administered systemic heparinization via intravenous injection of 45 IU unfractionated heparin per kg body weight. Diagnostic cerebral angiography and rotational angiography with three-dimensional (3D) reconstruction of the aneurysm was performed at the beginning of the procedure to determine a suitable working projection that clearly revealed the parent artery together with the aneurysm neck and sac without foreshortening or over-projection. The diameters of the neck and fundus of the target aneurysm were measured after calibrating the DSA system.

Patients were treated with either coil occlusion alone or with stent-assisted coiling based on the anatomy of the parent vessel and aneurysm. The individual treatment strategy and the selection of access products and procedures were at the discretion of the operator.

In our study, an Echelon 10 microcatheter (Medtronic PLC, Dublin, Ireland) was used most frequently for coil embolization of cerebral aneurysms. The Avenir® coil system includes numerous coil models that can be selected based on clinical needs (see Supplementary Material). For stent-assisted coil embolization, most operators used an Enterprise (Codman Neurovascular, Raynham, MA, USA) or LVIS^TM^ stent (MicroVention Inc., Tustin, CA, USA).

A microcatheter supported by a shaped microguidewire was inserted into the aneurysm or the parent artery under roadmap guidance. After determining the appropriate coil diameter and length based on the angiographic measurements, a suitable coil was placed into the aneurysm or the parent artery. This process was repeated until the interventionist deemed that sufficient occlusion was achieved. After coil occlusion, angiography was repeated at the previously identified working projection and at standard posterior-anterior and lateral projections to determine the extent of aneurysm occlusion as well as the patency of the parent artery.

The patients assigned to the Avenir group underwent a procedure in which Avenir® mechanically detachable coils (Wallaby Medical, Shanghai, China) were used. This device includes 3D framing coils, two-dimensional (2D) filling coils, 2D finishing coils, and 3D finishing coils. The 3D framing coils were used to form the initial basket within the target aneurysm. Avenir® filling coils were then used to fill the space within the framing coils and Avenir® finishing coils were used to complete the final occlusion at the aneurysm neck. The diameter and length of the Avenir® coils used for each procedure were chosen by the interventionist.

Patients assigned to the Axium group were treated with mechanically detachable Axium^TM^ coils (Medtronic PLC). The coil occlusion procedure and the decision-making criteria, including all technical details, were identical for both patient groups.

### Outcome Parameters

#### Efficacy

The primary outcome parameter was the degree of aneurysm occlusion as determined by a follow-up DSA that was performed 6 months after coil treatment. The quantitative analysis was based on the amount of contrast medium filling the parent artery and entering into the aneurysm. The Raymond-Roy Occlusion Classification (RROC) system was used to evaluate coiled aneurysms; the ratings included Class I, complete obliteration; Class II, residual neck; and Class III, residual aneurysm (19). The extent of aneurysm occlusion was evaluated by three qualified investigators who performed their assessments independently and who were not involved in the clinical trial. One investigator was based in China and two were from Germany.

#### Safety

Surgical complications of these procedures included hematoma at the puncture site, intracranial vessel perforation or dissection, aneurysm rupture, parent artery occlusion, incomplete filling of the aneurysm caused by the inability to reposition the microcatheter after it has been dislodged from the aneurysm, distal vessel occlusion due to emboli, local vasospasm, migration or dislocation of coils, coils that detached too early or failed to detach, and ischemic stroke.

### Follow-Up Examinations and Visits

All patients underwent a final DSA during the procedure immediately after coiling. Patients were then observed for the following 6 months. The patients were first evaluated 3 months post-procedure at the outpatient clinic or by telephone. Scheduled follow-up angiography was performed during the second follow-up 6 months after treatment. Any additional follow-up examinations that were required as clinical routine and/or specific patient needs were not included in the framework of this clinical trial.

### Sample Size

This study was designed as a non-inferiority trial. The aneurysm occlusion rate at 6 months follow-up based on angiography findings was assessed according to the RROC scheme (19, 20).

The clinically recognized threshold for non-inferiority was −10% (absolute value) based on equal numbers of patients in both the Avenir and Axium groups. With α set at 0.025 (unilateral) and the power of the test (1-β) set at 0.8, the sample size for the non-inferiority trial of each group was calculated at *n* = 129 (PASS 13 software; NCSS LLC, Kaysville, UT, USA). Taking into account a potential dropout rate of 20% and the need for randomization, we enrolled 324 patients for the entire study, with 162 subjects assigned randomly to each group. The clinical trial institutions were requested to pursue active patient enrollment; each was asked to enroll a maximum of 162 patients (50% of the total sample size) and a minimum of 24 patients (7% of the total sample size).

### Statistical Analysis

An independent data management center (Peking University First Hospital) was engaged to evaluate the clinical and angiography data and to perform the calculations and statistical analysis. SAS9.4 software (SAS Institute Inc., Cary, NC, USA) was used for the statistical analysis. Two-sided tests were used for all statistical comparisons unless specified. Differences between groups were deemed statistically significant for *p* < 0.05. Quantitative indicators were expressed as means, standard deviations (SDs), medians, minimums, maximums, and quartiles. Numerical data were expressed as frequencies (proportions). A paired *t*-test (in the event of homogeneous variance and normal distribution) or Wilcoxon rank-sum test was used to compare quantitative data between groups based on the distribution of data. A chi-square test or Fisher's exact test (if chi-square test was not applicable) was used to evaluate classified data. The Cochran–Mantel–Haenszel (CMH) test was used for ranked data.

## Results

### Baseline Characteristics of Patients

**Figure 1:**
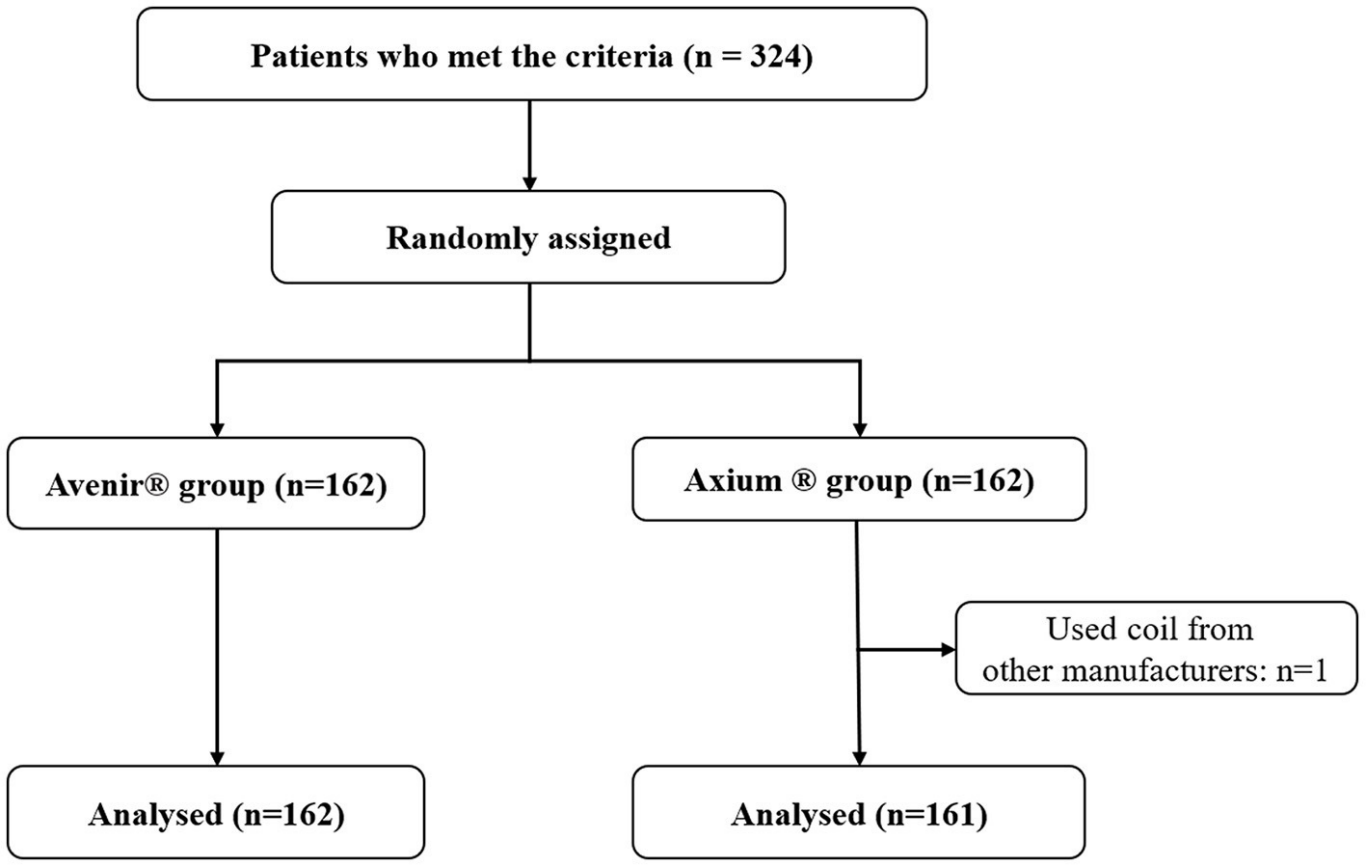
Flow chart documenting inclusion of patients in the study.

Three hundred and twenty-four patients were initially enrolled in the study. One patient in the Axium group was excluded because this coil was not used in the procedure. This resulted in a final total of 323 patients (Figure 1).

General and demographic information for the patients in each of the two groups is included in Table 1. A full set analysis (FAS) was performed on 162 patients assigned to the Avenir group and 161 patients assigned to the Axium group. At the 6-month follow-up visit, imaging studies were completed in 139 patients, which included 140 aneurysms diagnosed in patients assigned to the Avenir group and 142 aneurysms in patients assigned to the Axium group. The two groups were generally comparable; we observed no statistically significant differences in age, gender, ethnicity, pretreatment Hunt-Hess grade, or clinical data (*p* > 0.05). However, we did identify significant differences between the two groups with respect to past medical and surgical history (*p* < 0.05).

**Table 1:** Characteristics of patients assigned to the Avenir and Axium groups.

**Characteristics**	**Total**	**Avenir group** ***n* = 162**	**Axium group *n* = 161**	***p***
Age in years mean ± SD	56.90 ± 9.66	57.63 ± 8.45	56.16 ± 10.73	0.385
Gender Male/female	94/229	44/118	50/111	0.441
Ethnicity *n* (%)				0.549
Han Chinese	311 (96.28)	157 (96.91)	154 (95.65)	
Other	12 (3.72)	5 (3.09)	7 (4.35)	
Height in cm mean ± SD (*n)	163.2 ± 17.07	162.66 ± 6.99 (*5)	163.77 ± 7.13 (*8)	0.279
Has relevant past/current medical history *n* (%)	278 (86.07)	146 (90.12)	132 (81.99)	0.035
Past surgical history *n* (%)	62 (38.27)	37 (22.98)	99 (30.65)	0.003
Allergy history *n* (%)	13 (8.02)	14 (8.70)	27 (8.36)	0.828
Weight in kg mean ± SD (*n)	65.50 ± 10.92	65.31 ± 10.92 (*1)	65.69 ± 10.95	0.724
Diameter of the aneurysm in mm median (IQR) (*n)	5.35 (4.00–7.50)	5.60 (4.40–7.20) (*44)	5.10 (3.80–7.80) (*61)	0.259
Hunt-Hess grade *n* (%)				0.623
Grade 0	225 (69.66)	111 (68.52)	114 (70.81)	
Grade I	61 (18.89)	31 (19.14)	30 (18.63)	
Grade II	31 (9.60)	17 (10.49)	14 (8.70)	
Grade III	6 (1.86)	3 (1.85)	3 (1.86)	

The symbol * represents the missing sample size.

A total of 1,210 coils were successfully implanted into patients assigned to the Avenir group, resulting in an average of seven coils used for each aneurysm. By contrast, 973 coils, or an average of six coils per aneurysm, were used in procedures performed on patients in the Axium group.

There was also no significant difference between the two groups with respect to stent-assisted coiling. One hundred and nineteen patients (73.46%) assigned to the Avenir group were treated with stent-assisted therapy, compared to 117 (72.67%) in the Axium group.

### Efficacy Endpoints

**Figure 2:**
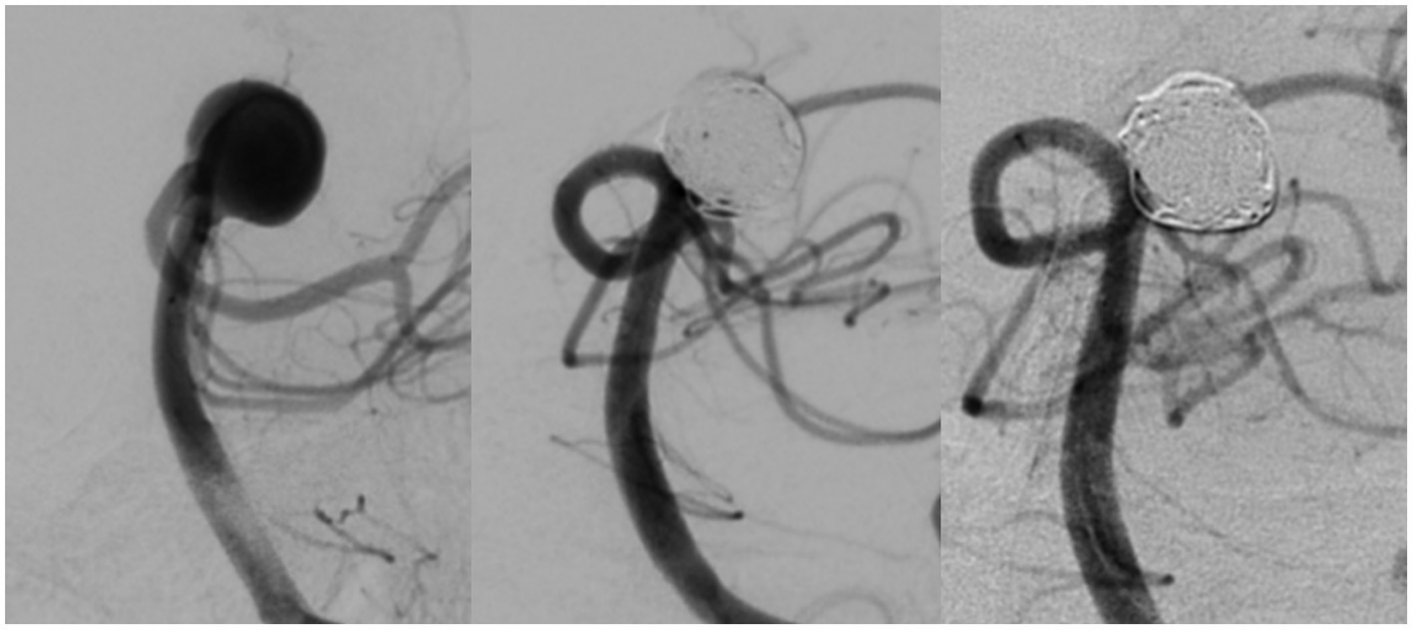
DSA images of an aneurysm at the basilar artery bifurcation before treatment **(left)**, immediately after coil occlusion with Avenir coils **(middle)**, and at 6-month follow-up **(right)**.

The aneurysm occlusion rates at 6 months post-procedure are shown in Table 2. One hundred and twenty-four of 131 aneurysms treated in patients in the Avenir group (94.66%) achieved an RROC classification of I or II; 127 of the 131 aneurysms treated in patients in the Axium group (96.95%) achieved this result. As indicated by these results, we identified no statistically significant differences in the occlusion rate between the two groups (*p* = 0.356). A representative case from the Avenir group is shown in Figure 2.

**Table 2:** Rates and characteristics of aneurysm occlusion at 6 months post-procedure.

**Characteristics, *n* (%)**	**Avenir group**	**Axium group**	***p***
	**(*n* = 131)**	**(*n* = 131)**	
Rate aneurysm occlusion			0.449
RROC I	110 (83.97)	113 (86.26)	
RROC II	14 (10.69)	14 (10.69)	
RROC III	7 (5.34)	4 (3.05)	
Some aneurysm occlusion			0.356
No (RROC III)	7 (5.34)	4 (3.05)	
Yes (RROC I or II)	124 (94.66)	127 (96.95)	
Complete aneurysm occlusion			0.603
No (RROC II or III)	21 (16.03)	18 (13.74)	
Yes (RROC I)	110 (83.97)	113 (86.26)	

### Safety Endpoints

We detected no significant differences in modified Rankin Scale scores 6 months after the endovascular coil procedure.

Surgical complications were recorded up until the end of the 6-month follow-up period (Table 3). There were no procedure-associated patient deaths in our study. Among the patients assigned to the Avenir group, one had a ruptured aneurysm, 22 had ischemic strokes, three exhibited incomplete aneurysm filling, and one developed a hematoma at the puncture site. In the Axium group, two patients had a ruptured aneurysm, 14 had ischemic strokes, two exhibited incomplete aneurysm filling, three developed hematomata at the puncture site, and one had difficulties with coil detachment. There were no statistically significant differences between the two groups with respect to these complications.

**Table 3:** Complications associated with the use of Avenir® or Axium^TM^ coils to treat aneurysms.

**Characteristics, *n* (%)**	**Avenir group**	**Axium group**	***p***
	***n* = 162**	***n* = 161**	
Surgical complications	27	22	0.645
Ischemic stroke	22	14	
Incomplete aneurysm filling	3	2	
Hematoma at the puncture site	1	3	
Aneurysm rupture	1	2	
Difficult coil detachment	0	1	

## Discussion

This study aimed to compare the efficacy and safety of the Avenir® and Axium^TM^ coils for the treatment of intracranial aneurysms. As this was a preliminary investigation, our study was designed to evaluate non-inferiority. We found that the Avenir® coil system was both safe and effective for the treatment of intracranial aneurysms and that it was not inferior to the Axium^TM^ coil system when used to promote occlusion of intracranial aneurysms.

The Axium^TM^ system used in this study was previously evaluated by Kim et al. (21) and presented promising results in terms of its efficacy and safety. This device used here is a bare platinum coil, which is generally preferred by many interventionists. Recent developments include several advanced coil materials, including polymer-coated matrix coils and hydrocoils with a hydrophilic gel surface. However, only a few large-scale studies have been performed to evaluate these coils. The general consensus is that there appears to be little difference in the outcomes compared to the use of bare platinum coils (22). However, Broeders et al. (23) reported that the rate of complete occlusion may be higher in procedures performed using coils made from these materials.

The mechanisms underlying recurrent aneurysm perfusion following coil occlusion have not been fully clarified. Various factors have been implicated in this complication including the precise location and size of the aneurysm, blood flow, blood pressure, previous incidences of aneurysm rupture, and the volume of coils used to fill the aneurysm (24–26). The coils must fill the aneurysm in a uniform and dense fashion in order to avoid recurrence (25, 27).

Several clinical studies and multicenter trials with long-term follow-up reported an aneurysm occlusion rate of 90–96% (28–30). In the present study, the 6-month follow-up DSA after coil treatment showed an aneurysm occlusion rate of up to 94.66% (i.e., RROC class I and class II) for patients assigned to the Avenir group and 96.95% for those in the Axium group. These results suggest that comparable rates of recurrence may also be expected over the long term.

Coil occlusion endovascular procedures carry the risk of both device-related and device-unrelated surgical complications. The former group includes hematoma at the puncture site, intracranial vessel perforation, aneurysm rupture, parent artery occlusion, embolic vessel occlusion, vasospasm, migration or dislocation of coils, ischemic stroke, early coil detachment or detachment failure, and neurological deficits resulting in disability or death. Procedure-related (but not device-related) complications were reported for 27 patients assigned to the Avenir group and 22 in the Axium group. These complications were primarily ischemic strokes that were diagnosed based on the symptoms and diffusion-weighted imaging (DWI) studies performed 24 h after embolization therapy. It is important to note that the ischemia that develops in response to vasospasm of a ruptured aneurysm may be difficult to distinguish from ischemia as a complication of intervention. Thus, all incidents of cerebral ischemia are counted as postoperative complications.

The main technological advance used by the Avenir® coil system is a novel detachment mechanism. This new mechanism is a noose-based and passive mechanically detachable coil system that does not require the use of any detachment tools. The forward-directed force of the coil system is based on the softness of the coils and the friction that develops between the pushrod and the microcatheter (11). The push rod of the Avenir® coil system has a tapered design that maintains stability at the proximal end and permits the distal end to turn smoothly. The detachment mechanism influences the microscopic movements of the distal ends of the conveyor rod and microcatheter, thereby reducing the pressure of the coils against the aneurysm wall. The Avenir® coil system is detached through a noose by manual breakage of a proximal push rod. We experienced a 100% success rate for coil detachment using the Avenir® mechanism. This mechanism ensures safety and limits the amount of time needed to manage peri-procedural aneurysm ruptures. If a coil does not undergo successful detachment, the distal end can become trapped or locked, and the coil may stretch, unwind, or even break. Once a coil becomes loose, it is difficult if not impossible to push it back into the aneurysm or to withdraw it completely into the microcatheter. Therefore, controlled coil detachment is critical for a successful endovascular procedure.

This study was designed as a preliminary, non-inferiority trial and therefore has some limitations. Further research will be needed to determine whether the new detachment method used in the Avenir® system improves the delivery of the coils. Longer follow-up will be needed to determine the risk of recurrence at 1 year and beyond.

## Conclusion

Our study revealed no apparent differences in short-term occlusion rates and safety in a comparison of endovascular procedures performed using the Avenir® vs. the Axium^TM^ detachable coil systems.

## Data Availability Statement

The raw data supporting the conclusions of this article will be made available by the authors, without undue reservation.

## Ethics Statement

The study was approved by the Ethics Committees of Beijing Tiantan Hospital of Capital Medical University, Xuanwu Hospital of Capital Medical University, Huashan Hospital of Fudan University, The First Hospital of Jilin University, The First Affiliated Hospital of Dalian Medical University, The First Affiliated Hospital of Harbin Medical University, Shanxi Provincial People's Hospital, Peking University International Hospital, The First Hospital of Shanxi Medical University, and Tangshan Worker's Hospital. The trial passed the Shanghai FDA inspection with no major findings and the trial also passed each sites' quality check according to the hospitals' clinical trial quality check. The clinical trial registration number is ChiCTR2100046506. The patients/participants provided their written informed consent to participate in this study.

## Author Contributions

WL and MY: conceptualization and design of the study, data collection, statistical analysis, organization and presentation of the data, review, and critique of the manuscript. AC and HH: data curation, evaluation of the rate of aneurysm occlusion, review, and editing of the manuscript. HW: organization and execution of the study, data collection, and critique of the manuscript. XX: analyzing data and critique of the manuscript. YG: data interpretation and critical review of the manuscript. HS: clinical study design and patient recruitment. HJ: patient management, follow-up design, and conceptualization of the study. FW: manuscript editing and critique of the manuscript. YZ: acquisition, analysis and interpretation of data, and technical and administrative support. GG: concept, design, and drafting of the manuscript. HZ and YL: design of the study, writing of the first draft, review, and critique of the manuscript. All authors have read and agreed to the published version of the manuscript.

## Conflict of Interest

HH is co-founder and share-holder of phenox GmbH, which is selling Avenir coils in Europe and the US. The remaining authors declare that the research was conducted in the absence of any commercial or financial relationships that could be construed as a potential conflict of interest.

## Publisher's Note

All claims expressed in this article are solely those of the authors and do not necessarily represent those of their affiliated organizations, or those of the publisher, the editors and the reviewers. Any product that may be evaluated in this article, or claim that may be made by its manufacturer, is not guaranteed or endorsed by the publisher.
