# Supplementary material for: Avenir® vs. AxiumTM Coils for the Treatment of Intracranial Aneurysms: Results of a Multicenter Randomized Controlled Trial With Short-Term Follow-Up
Source: Front Neurol. 2022 Jan 26;12:817989. doi: 10.3389/fneur.2021.817989 (PMC8825471; doi:10.3389/fneur.2021.817989)
Supplement: Supplementary file 1 [file Table_1.DOCX]

Supplement 1: Avenir^®^ Coil Specification Sheet

| 18 Series - 3D  framing coil | | | | 10 Series - 3D  framing coil | | | | 10 Series – 2D  filling coil | | | 10 Series – 3D  finishing coil | | | 10 Series – 2D  finishing coil | | | |
| --- | --- | --- | --- | --- | --- | --- | --- | --- | --- | --- | --- | --- | --- | --- | --- | --- | --- |
| D (mm) | L (cm) | WD (mm) | CD (mm) | D (mm) | L (cm) | WD (mm) | CD (mm) | D (mm) | L (cm) | WD (mm) | D (mm) | L (cm) | WD (mm) | D (mm) | L (cm) | WD (mm) | CD (mm) |
| 6.0 | 16 | 0.32 | 4.50 | 2.0 | 2 | 0.28 | 2.00 | 3.0 | 4 | 0.28 | 1.0 | 2 | 0.28 | 1.0 | 2 | 0.28 | 1.00 |
| 7.0 | 19 | 0.32 | 5.25 | 2.0 | 3 | 0.28 | 2.00 | 3.0 | 6 | 0.28 | 1.5 | 1 | 0.28 | 1.5 | 2 | 0.28 | 1.50 |
| 8.0 | 27 | 0.34 | 6.00 | 2.0 | 6 | 0.28 | 2.00 | 3.0 | 8 | 0.28 | 1.5 | 2 | 0.28 | 1.5 | 3 | 0.28 | 1.50 |
| 9.0 | 30 | 0.34 | 6.75 | 2.5 | 5 | 0.28 | 2.50 | 3.0 | 10 | 0.28 | 1.5 | 3 | 0.28 | 1.5 | 4 | 0.28 | 1.50 |
| 10.0 | 34 | 0.34 | 7.50 | 3.0 | 4 | 0.28 | 3.00 | 4.0 | 4 | 0.28 | 1.5 | 4 | 0.28 | 2.0 | 2 | 0.28 | 2.00 |
| 11.0 | 37 | 0.37 | 8.25 | 3.0 | 6 | 0.28 | 3.00 | 4.0 | 6 | 0.28 | 1.5 | 6 | 0.28 | 2.0 | 3 | 0.28 | 2.00 |
| 12.0 | 40 | 0.37 | 9.00 | 3.5 | 6 | 0.28 | 3.50 | 4.0 | 8 | 0.28 | 2.0 | 1 | 0.28 | 2.0 | 4 | 0.28 | 2.00 |
| 13.0 | 43 | 0.37 | 9.75 | 4.0 | 7 | 0.28 | 3.60 | 4.0 | 10 | 0.28 | 2.0 | 2 | 0.28 | 2.0 | 6 | 0.28 | 2.00 |
| 14.0 | 47 | 0.37 | 10.50 | 4.0 | 13 | 0.28 | 3.60 | 4.0 | 15 | 0.28 | 2.0 | 3 | 0.28 | 2.5 | 3 | 0.28 | 2.50 |
| 15.0 | 50 | 0.37 | 11.25 | 5.0 | 8 | 0.28 | 3.75 | 4.0 | 20 | 0.28 | 2.0 | 4 | 0.28 | 2.5 | 4 | 0.28 | 2.50 |
| 16.0 | 50 | 0.37 | 12.00 | 5.0 | 17 | 0.28 | 3.75 | 5.0 | 10 | 0.28 | 2.0 | 6 | 0.28 | 3.0 | 4 | 0.28 | 3.00 |
| 18.0 | 50 | 0.37 | 13.50 | 6.0 | 11 | 0.28 | 4.50 | 5.0 | 15 | 0.28 | 2.0 | 8 | 0.28 | 3.0 | 6 | 0.28 | 3.00 |
| 20.0 | 50 | 0.37 | 15.00 | 6.0 | 20 | 0.28 | 4.50 | 5.0 | 20 | 0.28 | 2.5 | 3 | 0.28 | 3.0 | 8 | 0.28 | 3.00 |
| 22.0 | 50 | 0.37 | 16.50 | 7.0 | 13 | 0.28 | 5.25 | 6.0 | 10 | 0.28 | 2.5 | 4 | 0.28 | 3.0 | 10 | 0.28 | 3.00 |
| 25.0 | 50 | 0.37 | 18.75 | 7.0 | 24 | 0.28 | 5.25 | 6.0 | 15 | 0.28 | 2.5 | 6 | 0.28 | 4.0 | 6 | 0.28 | 3.60 |
| 25.0 | 60 | 0.37 | 18.75 | 8.0 | 16 | 0.28 | 6.00 | 6.0 | 20 | 0.28 | 3.0 | 4 | 0.28 | 4.0 | 8 | 0.28 | 3.60 |
|  | | | | 8.0 | 27 | 0.28 | 6.00 | 7.0 | 15 | 0.28 | 3.0 | 6 | 0.28 | 4.0 | 10 | 0.28 | 3.60 |
|  |  |  |  | 9.0 | 23 | 0.28 | 6.75 | 7.0 | 20 | 0.28 | 3.0 | 8 | 0.28 | 5.0 | 8 | 0.28 | 3.75 |
|  |  |  |  | 9.0 | 30 | 0.28 | 6.75 | 7.0 | 30 | 0.28 | 3.0 | 10 | 0.28 | 5.0 | 10 | 0.28 | 3.75 |
|  |  |  |  | 10.0 | 27 | 0.28 | 7.50 | 8.0 | 20 | 0.28 | 3.5 | 4 | 0.28 |  | | | |
|  |  |  |  | 10.0 | 34 | 0.28 | 7.50 | 8.0 | 30 | 0.28 | 3.5 | 6 | 0.28 |  |  |  |  |
|  |  |  |  |  | | | | 9.0 | 30 | 0.28 | 3.5 | 8 | 0.28 |  |  |  |  |
|  |  |  |  |  |  |  |  | 10.0 | 30 | 0.28 | 4.0 | 6 | 0.28 |  |  |  |  |
|  |  |  |  |  |  |  |  |  | | | 4.0 | 8 | 0.28 |  |  |  |  |
|  |  |  |  |  |  |  |  |  |  |  | 4.0 | 10 | 0.28 |  |  |  |  |
|  |  |  |  |  |  |  |  |  |  |  | 5.0 | 6 | 0.28 |  |  |  |  |
|  |  |  |  |  |  |  |  |  |  |  | 5.0 | 8 | 0.28 |  |  |  |  |
|  |  |  |  |  |  |  |  |  |  |  | 5.0 | 10 | 0.28 |  |  |  |  |
|  |  |  |  |  |  |  |  |  |  |  | 6.0 | 6 | 0.28 |  |  |  |  |
|  |  |  |  |  |  |  |  |  |  |  | 6.0 | 8 | 0.28 |  |  |  |  |
|  |  |  |  |  |  |  |  |  |  |  | 6.0 | 10 | 0.28 |  |  |  |  |

D; diameter; L, length; WD, wire diameter; CD, coil diameter.
